# Fracture Toughness of Moldable Low-Temperature Carbonized Elastomer-Based Composites Filled with Shungite and Short Carbon Fibers

**DOI:** 10.3390/polym14091793

**Published:** 2022-04-27

**Authors:** Semen D. Ignatyev, Eugene S. Statnik, Dmitriy Yu. Ozherelkov, Dmitry D. Zherebtsov, Alexey I. Salimon, Dilyus I. Chukov, Victor V. Tcherdyntsev, Andrey A. Stepashkin, Alexander M. Korsunsky

**Affiliations:** 1 Laboratory of Functional Polymer Materials, NUST MISiS, 119049 Moscow, Russia; ignatyev.s.11@gmail.com (S.D.I.); dmitry_zherebtsov@bk.ru (D.D.Z.); a.salimon@skoltech.ru (A.I.S.); dil_chukov@mail.ru (D.I.C.); a.stepashkin@misis.ru (A.A.S.); 2 HSM Laboratory, Center for Energy Science and Technology, Skoltech, 121205 Moscow, Russia; eugene.statnik@skoltech.ru; 3 Catalysis Laboratory, NUST MISIS, 119991 Moscow, Russia; d.ozherelkov@gmail.com; 4 MBLEM, Department of Engineering Science, University of Oxford, Oxford OX1 3PJ, UK; alexander.korsunsky@eng.ox.ac.uk

**Keywords:** composites, rubbers, carbonization, shungite, short carbon fibers, fracture toughness, stress intensity factor, three-point bending test, acoustics, microstructure

## Abstract

This work evaluated the fracture toughness of the low-temperature carbonized elastomer-based composites filled with shungite and short carbon fibers. The effects of the carbonization temperature and filler content on the critical stress intensity factor (*K*_1*c*_) were examined. The *K*_1*c*_ parameter was obtained using three-point bending tests for specimens with different *l*/*b* ratio (notch depth to sample thickness) ranging from 0.2 to 0.4. Reliable detection of the initiation and propagation of cracks was achieved using an acoustic sensor was attached to the samples during the bending test. The critical stress intensity factor was found to decrease linearly with increasing carbonization temperature. As the temperature increased from 280 to 380 °C, the *K*_1*c*_ parameter was drastically reduced from about 5 to 1 MPa·m^1/2^ and was associated with intense outgassing during the carbonization step that resulted in sample porosity. The carbon fiber addition led to some incremental toughening; however, it reduced the statistical dispersion of the *K*_1*c*_ values.

## 1. Introduction

Nowadays, polymer-matrix composite materials can be fabricated by incorporating different reinforcing components (fibers, powders, etc.) in a polymer (e.g., elastomeric) binder. Successful material combinations demonstrate optimal sets of properties in terms of strength, stiffness, fracture toughness and fatigue resistance [1].

Composite materials filled with carbon fillers are widely used in modern industry and are an important subject of ongoing research [2,3]. Recently, a new class of composite materials emerged based on carbonized polymer matrices filled with carbon fillers. Elastomer carbonation is a complex of thermochemical reactions occurring in a polymer under the influence of a temperature-time field. The result of this process is the enrichment of the initial product with carbon, with simultaneous restructuring of its structure. On the quantitative side, carbonation leads to the production of a product, ideally consisting entirely of absolutely pure carbon. On the qualitative side, the polymer turns into one of the most stable carbon compounds, such as graphite or diamond.

The main advantage of using the carbonization process for various polymers is a significant increase in a number of their properties, in particular, an increase in strength. Structural changes occurring during carbonation also lead to an increase in the thermal properties of materials and their stable behavior when exposed to thermal fields.

Control over the production schedules allows obtaining different properties. For instance, it is possible to improve selectively or summarily the mechanical, thermal, electrical, and tribological properties of the composites [4,5]. The key feature of these composite materials is their manufacturing route that includes three main sequential processing stages: component mixing, vulcanization and low-temperature carbonization. The first two stages allow creating the desired shape of the product via the use of the necessary die shapes while the final carbonization stage sets the product final geometry that can be achieved considering shrinkage. The manufacturing history determines the combination of the final physical, mechanical and electrical properties.

The strength properties of composite materials are closely connected with the degree of bonding between the reinforcing phase and the matrix, and the homogeneity of the resulting material. When composites with carbonized matrices are considered, the key factor determining the mechanical properties are the temperature regimes for composite carbonization. The homogeneity parameter plays an important role in service performance, as it determines the product’s resistance to crack initiation and propagation [6].

The vital drawback of the vulcanization and carbonization stages is the creation of internal stresses that affect the fracture resistance of the end product. The principal criterion of the fracture toughness is the stress intensity factor (*K*_1*c*_ for Mode I). Hence, the evaluation of this parameter is required for such composites. The fracture toughness parameter defines the ability of the material to resist the growth of an already existing crack, and thus relates to material durability. Ensuring durability is essential throughout fabrication, including final cutting. For instance, the fabrication of bipolar plates for fuel cells requires the use of milling with a diamond cutter to form flow channels supplying molten salts. Machining low toughness material may leave defects that promote fracture and lead to major financial and operational losses [7].

### Fracture Toughness Evaluation

Most common approaches to fracture toughness evaluation in materials tend to consider Mode I (opening mode). However, polymer composite materials often fail by inter-layer shear [3,8,9], so Mode II and Mode III testing become significant.

Furthermore, most studies of graphite fracture toughness are devoted to the determination of the static fracture toughness [10,11,12,13,14]. The determination of the dynamic fracture toughness has not been studied so widely [15]. The approaches to determine the fracture toughness include:

1. Theoretical calculations recommended by ASTM are based on significant simplifying simplifications [15] and therefore deliver estimates of limited reliability. The approach is relatively straightforward to implement but assessment quality relies crucially on high precision in crack length evaluation that in practice can be difficult to achieve, particularly in dynamic processes.
2. The displacement or strain field method that performs fracture toughness evaluation based on the displacement (or strain) field around the crack tip [16]. This option involves a large number of data collection, interpretation and computation operations. Some parameters must be adjusted for specific experiment and may lead to the emergence of systematic errors.
3. The J-integral method [17] is another parameter that describes the energy required for crack propagation that is related to fracture toughness. Minimizing the error of the J-integral determination requires carry out a sufficiently large series of experiments. In addition, the values of the J-integral calculated by different methods often differ significantly [18].
4. The crack tip opening displacement method [19] is effective only under conditions of linear elasticity.
5. The critical crack tip opening angle method [20]. This method requires to measure simple geometry like opening crack angle from the captured image. In practice, the opening crack angle is usually determined manually, which also can create significant artificial errors.

In this study, the fracture toughness of low-temperature carbonized elastomer-based composites filled with shungite and short carbon fibers was investigated. The impact of the carbonization temperature and carbon fiber content on the stress intensity factor was analyzed. The mechanical behavior of such composites is very similar to the mechanics of graphite that fractures in a brittle manner without plastic deformation. The novelty and scientific soundness of this investigation are based on the following key points: (a) it was methodologically showed how to prepare and test carbonized highly filled elastomers in fast way; (b) it was statistically proven that a single *l/b* ratio is appropriate for stress intensity factor estimation, i.e., no need to prepare different *l/b* ratios; (c) the *K*_1*c*_ parameter for such class of materials are not previously studied according to the literature search made during article writing. Moreover, observed outcomes disclose cause and effect relationships between structure and properties that will be studied in further article.

## 2. Materials and Methods

### 2.1. Specimens Preparation

The matrix of composite materials was based on BNKS-18 AMN TU 38.30313-2006 nitrile butadiene rubber (NBR) that was supplied from JSC “Krasnoyarsk Synthetic Rubber Plant” (Krasnoyarsk, Russia) with a 17–20 wt.% of the acrylonitrile and 0.4 wt.% of the ash content, respectively. The matrix was filled with the following components: shungite filler Carbosil T-20 (TU 5716-004-75625634-2006) (Ecochim LLC, Saint Petersburg, Russia) and with/without chopped fibers FibArm Fiber C (HC Composite, Moscow, Russia). A description of the compositions used in this study is summarized in Table 1.

The technology of composite materials manufacturing involves three stages. The first step is mixing and rolling of a matrix with the fillers by using BL-6175-A rubber mixing rollers (Dongguan Baopin Precision Instrument Co., Dongguan, China). The second step is the vulcanization of the prepared elastomer, which was carried out at a constant temperature of 170 °C during 10 min inside an AVPM-901 vulcanization press (Tesar Engeneering Ltd., Saratov, Russia) under a mold constant clamping force of 5 MPa. In order to create spatial grid into elastomer matrix, a dicumyl peroxide with a linear formula of C_18_H_22_O_2_ (CAS Number 80-43-3, Sigma-Aldrich Corp., St. Louis, MO, USA) was used as a vulcanizing agent (3 mass parts per hundred mass parts rubber for each compound). The final stage is low-temperature carbonization that was performed via a PM-16M muffle furnace (Electropribor LLC, Saint Petersburg, Russia) under an argon atmosphere.

**Figure 1:**
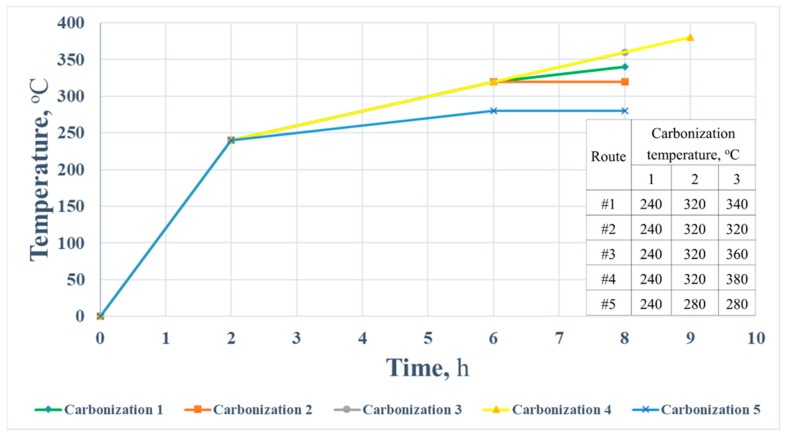
The scheme of applied regimes for the specimen’s carbonization.

In this study, the first and second stages were the same for all specimens, while the final stage was different. The applied variations in the carbonization are shown in Figure 1. The samples were made from three different compositions underwent each carbonization separately. The total time required for one carbonation was about 8 h.

### 2.2. Three-Point Bending Test and Acoustic Measurements

**Figure 2:**
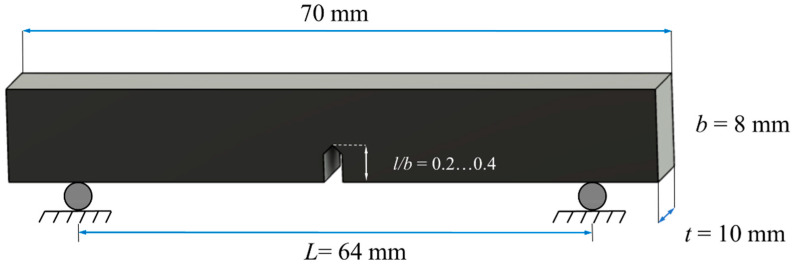
The shape and dimensions of specimens with the indicated range of *l/b* ratio.

Three-point bending test was performed using universal testing machine Zwick/Roell Z020 (Zwick GmbH, Ulm, Germany) adopted with a MultiXtens contact sensor to precisely record material displacement and strain. All specimens were conditioned before tests under the next conditions: standard 23/50 atmosphere during 88 h according to the ISO 291:2008. The traverse speed was 2 mm/min. The number of specimens with the same *l/b* ratio was ranged from 5 to 8. The geometry and shape of the sample are illustrated in Figure 2.

To understand and track crack initiation and propagation, an acoustic emission sensor was attached to the specimens. The monitoring characteristics were set up using a software package based on the PXI platform of National Instrument. The type of acoustic emission transducer was GT301, sensitivity of the transducer was 64 V/(m/s), operating frequency range was 50–1000 kHz, resonance frequency was 60 kHz. Parameters of data monitoring using acoustic emission were the following: threshold of 7 mV, data recording rate of 5 M, recording time of 5 ms, preamplifier gain of 20 dB. The obtained AE-meters were acted as indicators displaying the moment of crack initiation and propagation during the mechanical test. The obtained acoustic data was aligned and compared with standard force-displacement curve.

### 2.3. Stress Intensity Factor Evaluation

In general, the stress intensity factor depends on the applied stress, crack size, and the geometry:

(1) $$K_{1c}=\gamma \sigma \sqrt{\pi a},\;$$

where *γ* is so-called geometry factor defining the geometry of a crack system in relation to the applied load. Usually, this geometry factor can be looked up in technical reference books. For instance, for a centre crack in an infinite plate *γ* = 1.0. The geometry of the cracked body imposes an effect on the new crack tip stress field, thus modifying the value of the stress intensity factor. In general, if the edge crack is situated in a strip of finite width, w, then the correction factor becomes *γ = f*(*l*/*b*)**. The determination of this geometry term is a problem of stress analysis. Any realistic geometry requires recourse to numerical methods, as very few closed form solutions exist. The most popular and efficient method is finite element analysis. Other techniques include experimental and semi-theoretical; more will be said about this later.

In this research, stress intensity factor (*K*_1*c*_) was selected as the main parameter of fracture toughness evaluation. It was calculated using the following expression:

(2) $$K_{1C}=\gamma \frac{3PL\sqrt{\pi l}}{2tb^{2}},$$

where $\gamma =1.96-2.75\left(\frac{l}{b}\right)+13.66{\left(\frac{l}{b}\right)}^{2}-23.98{\left(\frac{l}{b}\right)}^{3}+25.22{\left(\frac{l}{b}\right)}^{4}$ [21]

P—the maximum load, N;
t—thickness, mm;
b—width, mm;
L—distance between supports, mm;
l—crack length, mm.

### 2.4. Statistical Analysis

Statistical data processing was determined by using the SPSS statistics software package [22]. To analyze the significance level (*p*-value) of the obtained *K*_1*c*_ values depending on *l/b* value, method of multiple comparisons with the control group using Dunnett’s criterion and one-way analysis of variance (ANOVA) were used.

### 2.5. Scanning Electron Microscopy

The microstructure of specimens after the three-point bending test was characterized by scanning electron microscope (SEM) Tescan VEGA Compact. Preliminary, samples were coated with antistatic to provide better conductivity. The images were collected using a secondary electrons (SE) detector with an accelerating voltage of 10 kV and a current of 300 pA.

## 3. Results and Discussions

### 3.1. Statistically Significant Estimations of K_1c_ Values

The results of determining the stress intensity factor for the three different compositions are presented in Table 2. To understand the dependence between *l/b* and *K*_1*c*_ parameters, the calculation was performed for the specimens undergoing #1 and #2 carbonization routes. The evaluation of the obtained values clearly indicates a slight difference between the determined values of *K*_1*c*_ for two different heat treatment modes. Limit calculated values ranged from 2.5 to 4.2 MPa·m^1/2^ and are main relevance to graphite and ceramic materials. The outputs demonstrate that the compositions filled with carbon fibres (CF25 and CF50) have higher values of stress intensity factor in comparison with the composition without carbon fibre addition (CF0). This effect is due to the strengthening property of carbon fiber. At the same time, increasing of the carbon fiber content from 25 (CF25) to 50 (CF50) mass parts does not give a significant growth in the *K*_1*c*_ parameter.

To analyze correlation of obtained stress intensity factor values depending on *l/b* ratio, one-way analysis of variance was used. It was assumed that the significance level is 0.95, then the *p*-value is 0.05. The null hypothesis was taken as the following: the *K*_1*c*_ parameter significantly varies when the *l/b* ratio is changed. In this case, if the calculated *p*-value will greater than 0.05, the null hypothesis will be rejected. The results of the one-way ANOVA are summarized in Table 3. For each composition the obtained *p*-value exceeded the specified level of 0.05. This implies that there is no dependence of the *K*_1*c*_ values on the *l/b* parameter. To conclude, the achieved result approves homogeneous character of cracks growth in the prepared composite materials regardless to their original size.

The Dunnett’s test was used as alternative statistical model for reliability. All mean *l/b* values were compared with the control group, where *l/b* was taken as 0.2. Significance level and *p*-value parameters were the same as for the one-way ANOVA. The results of the Dunnett’s test are shown in Table 4. The calculated pairwise comparison of mean *l/b* values also confirmed that the stress intensity factor is independent from the crack length.

The statistical analysis approved that it is sufficient to use a single *l/b* value for the *K*_1*c*_ parameter evaluation. For this reason, further processing was carried out for specimens with the *l/b* = 0.3. The obtained *K*_1*c*_ values with *l/b* = 0.3 for the carbonization routes #3–#5 are presented in Table 5. The stress intensity factor for the samples prepared via #3 and #4 carbonization routes is significantly lower than for specimens followed the #1, #2 and #5 carbonization regimes, respectively. This drop is connected with the impact of critical exposure temperatures on the elastomeric binder, which leads to a destruction of the cross-linked macromolecules of rubber matrix. The most stable behavior of fracture toughening is observed for the compositions undergoing #5 carbonization route. Such behavior is related with the lowest carbonization temperature that causes the minimum degree of destruction of the composite structure during heating and creates the most stable composition.

**Figure 3:**
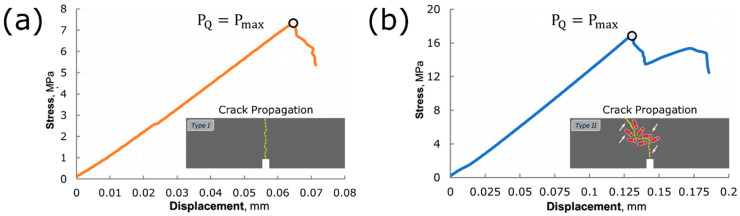
Typical stress-displacement curves obtained during bending test for stress intensity factor *K*_1*c*_ evaluation for CF0, CF25 and CF50 compositions: (**a**) type I, (**b**) type II.

The typical stress-displacement curves obtained for specimens without and with carbon fibres content are shown in Figure 3a and Figure 3b, respectively. The specimens were always broken at the elastic region. For composites containing only carbosil particles in the elastomeric matrix, the cracks were propagated along a straight trajectory with minimal distortions due to the localization of stresses on discrete carbosil particles as shown in the inset of Figure 3a. In contrast, for compositions containing carbon fibres, the cracks propagation was occurred along curvilinear trajectories. In this case, we assume that the carbon fibres reflect and redistribute obtained stresses as shown in the inset of Figure 3b, respectively.

**Figure 4:**
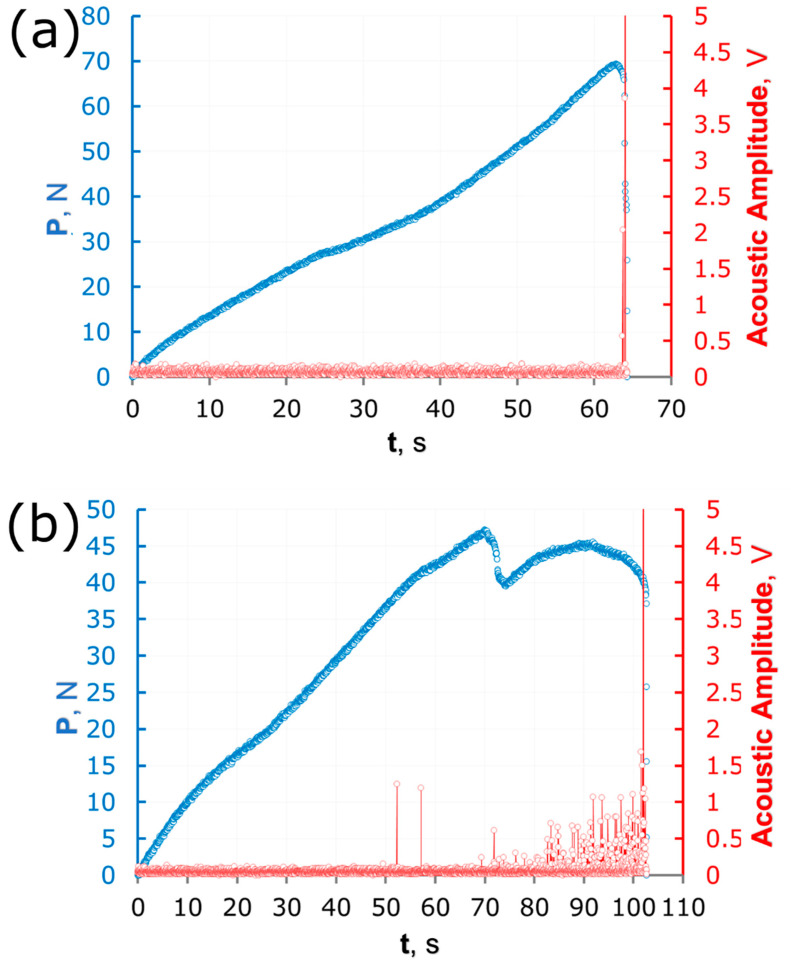
Typical plots determined by simultaneous bending test and acoustic measurements for (**a**) type I and (**b**) type II.

The moment of crack initiation and propagation was controlled by acoustic sensor synchronized with loading device. The typical synchronized load-time and acoustic amplitude-time curves for specimens without and with carbon fibre content are shown in Figure 4. The acoustic signal of the specimens with a CF0 composition (Figure 4a) demonstrates that the moment of crack initiation corresponds to the ultimate load to which the sample was withstand. No crack propagation up to the moment of fracture was noticed. In contrast, specimens containing carbon fibres (Figure 4b) showed another acoustic response. The first significant point in range between 50 and 60 s is connected with crack initiation while the amplitude peak at about 73 s corresponding to the ultimate load associates with crack and carbon fibre collision. Further behavior of the curve describes the process of crack growth. It is necessary to mention that the spatial orientation of fibres plays key role and directly influences the mechanical behavior of the composite since carbon fibres delay the propagation of the structure defect (in this case, it was crack).

**Figure 5:**
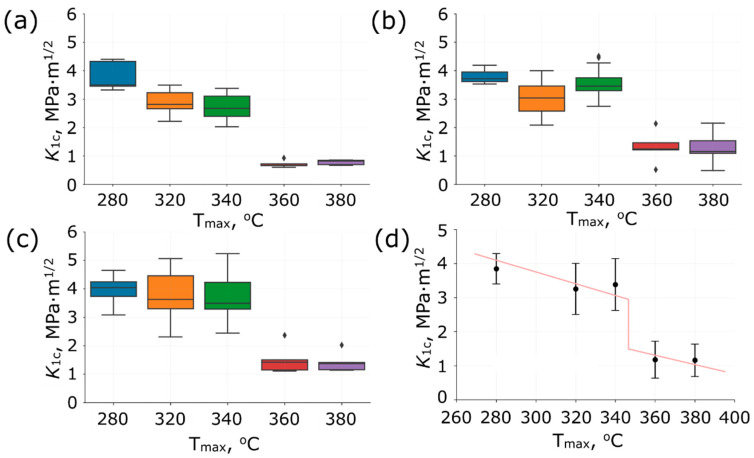
The relationship between stress intensity factor over maximum carbonization temperature for (**a**) CF0, (**b**) CF25, (**c**) CF50 compositions, and (**d**) Heaviside function fit for all data. The points on boxplots (**a**–**c**) represent outliers.

The relationship between stress intensity factor over maximum carbonization temperature is presented in Figure 5 using box plots. The compositions produced under maximum carbonization temperature of 280 °C have higher *K*_1*c*_ values among other temperatures. The growth of carbonization temperature provides the decreasing of *K*_1*c*_ values till 340 °C and dramatically drop after as shown in Figure 5d. This fall is related to a high degree of polymer binder degradation because two main chemical reactions are occurred during carbonization process promoting the formation of the final properties, namely, (a) the consolidation reaction and (b) the reaction of thermal-oxidative degradation. This point must be in mind when polymer composites are produced. The manufacturer should minimize the contribution of thermal-oxidative degradation reaction during carbonization. Therefore, a heating rate, holding time and limiting temperature of heating are crucial parameters for obtaining composite materials by such technology. The studies have shown that there is an active evolution of the gas phases during the carbonization process [23]. The intense gas formation surely influences the characteristics of the composite forming defects and porosity inside material structure.

**Figure 6:**
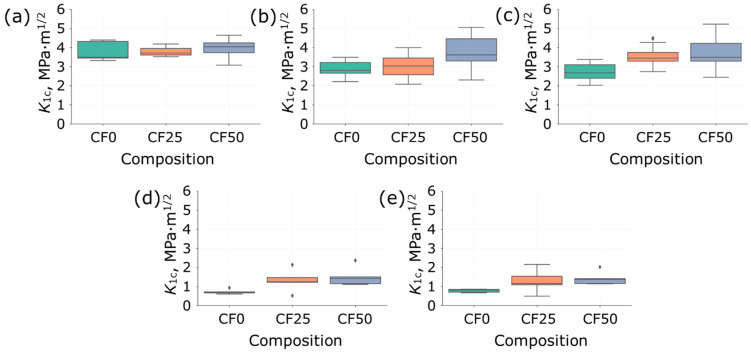
The relationship between stress intensity factor over different compositions for various maximum carbonization temperatures: (**a**) 280 °C, (**b**) 320 °C, (**c**) 340 °C, (**d**) 360 °C, and (**e**) 380 °C. The points on boxplots represent outliers.

The evaluations of the calculated *K*_1*c*_ values between different compositions produced with the same carbonization temperature are shown in Figure 6. The specimens carbonized at a maximum temperature of 280 °C have almost identical *K*_1*c*_ values in range from 3 to 5 MPa·m^1/2^ for all compositions. In contrast, an increasing trend in *K*_1*c*_ values has been observed for the compositions prepared under carbonization temperatures of 320 and 340 °C with the addition of carbon fibres. The samples made under maximum carbonization temperatures of 360 and 380 °C showed that such heating options lead to composite degradation and are not relevant. In this case, the maximum limiting threshold for *K*_1*c*_ values was 2 MPa·m^1/2^, which is significantly lower than the results of the other carbonizations.

The general summary of this experiment allows us to conclude about observed strengthening effect during carbon fibres addition. This fact can be explained via the crack propagation inhibition effect, which will provide products durability. On the other hand, the carbonization temperature of 280 °C allows to achieve the most optimal properties due to low degree of rubber matrix degradation with high carbon residue.

### 3.2. Microstructure Characterization and Fractography

**Figure 7:**
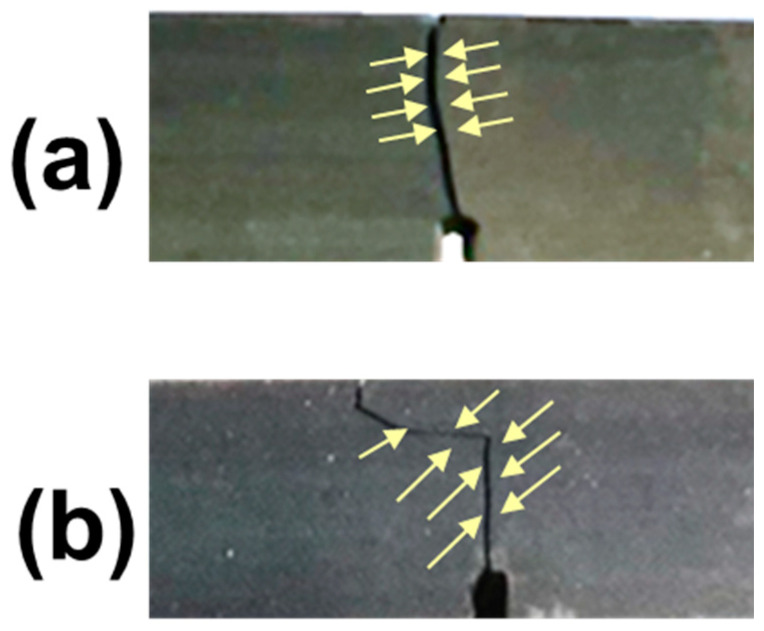
The examples of cracks propagation for true specimens: (**a**) type I and (**b**) type II.

**Figure 8:**
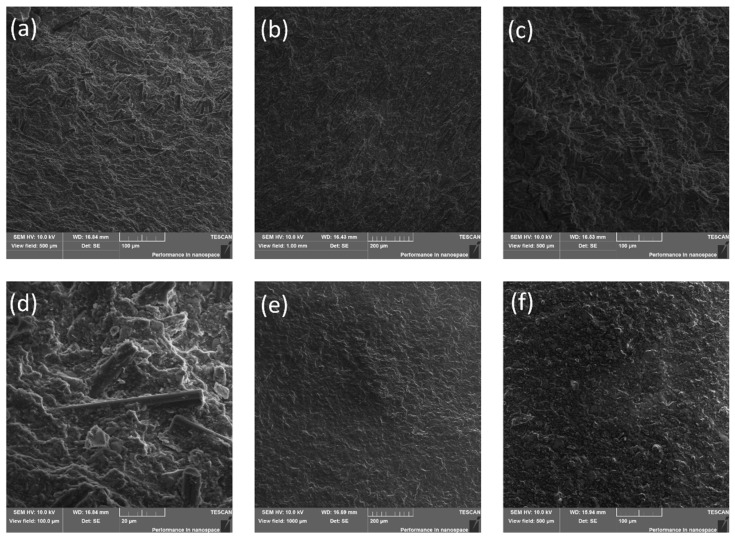
The microstructure of specimens (**a**–**d**) with carbon fibres content and (**e**,**f**) without them.

The macro view of fractured specimens without and with carbon fibres addition are illustrated in Figure 7a and Figure 7b, respectively. The cross-section microstructure for these specimens are shown in Figure 8. The specimens with carbon fibres content demonstrated multidirectional fibres orientation into polymer matrix. For instance, the area near crack initiation (Figure 8a) and at the centre (Figure 8b) showed preferred carbon fibres orientation close to 90°, in contrast, the location near the end of the crack (Figure 8c) showed 0° preferred orientation of carbon fibres distribution. Figure 8d illustrates a several carbon fibres appearance embedded into polymer matrix after fracture while Figure 8e,f demonstrated homogeneous microstructure of the samples without carbon fibres addition.

## 4. Conclusions

In this study, the fracture toughness behavior of the low-temperature carbonized elastomer-based composites filled with shungite and short carbon fibers were investigated. The impacts of the carbonization temperature and composition, namely, carbon fibres content, on the stress intensity factor (*K*_1*c*_) were examined. It was determined that

1. the change of *l/b* value is not statistically significant, and to determine *K*_1*c*_ values, it is sufficient to choose one *l/b* ratio.
2. the stress intensity factor of such composites inversely depends on the maximum carbonization temperature. The variation of obtained *K*_1*c*_ values ranged from 1 to 5 MPa·m^1/2^, which are most typical for brittle materials such as graphite or ceramics. The highest values of stress intensity factor were achieved by compositions carbonized at a maximum temperature of 280 °C.
3. the addition of carbon fibres to the composite material does not significantly increases the crack resistance of the composite.

To conclude, further studies of such composite materials are needed. The pathway of outlined plans will be related to the determination of the internal stresses originating inside composite during its production and strongly influencing its mechanical properties. Another complicated task is to find appropriate method for internal stresses relaxation that will allow the formation of composite structure most resistant to the mechanical influences. This section is not mandatory but can be added to the manuscript if the discussion is unusually long or complex.

## Figures and Tables

**Table 1:** The description of used compositions.

Composition	Carbosil T-20	Carbon Fibers
CF0	300	0
CF25	275	25
CF50	250	50

*Notes*: given numbers describe the mass parts per hundred mass parts rubber of the mixture component.

**Table 2:** Average values of stress intensity factor *K*_1*c*_ [MPa·m^1/2^] for specimens with different *l/b* ratio prepared by #1 and #2 carbonization routes.

Composition	Carbonization 1				Carbonization 2			
	*l/b*				*l/b*			
	0.2	0.3	0.4	0.5	0.2	0.3	0.4	0.5
CF0	2.88 ± 0.48	2.57 ± 0.52	2.95 ± 0.27	2.36 ± 0.16	2.91	3.16 ± 0.41	2.74 ± 0.56	2.61 ± 0.28
CF25	4.20 ± 0.48	3.55 ± 0.64	3.19 ± 0.21	3.52 ± 0.05	2.72 ± 0.12	3.17 ± 0.28	3.62 ± 0.34	2.41 ± 0.14
CF50	3.80 ± 0.45	4.22 ± 0.78	3.30 ± 0.08	3.38 ± 1.10	3.88 ± 0.36	4.11 ± 0.97	4.06 ± 0.91	3.39 ± 0.70

**Table 3:** The results of *p*-value estimation using the one-way ANOVA.

Composition	One-Way ANOVA
	*p*-Value (between Groups)
CF0	0.40
CF25	0.77
CF50	0.19

*Notes:* fixed factor: *l/b*; dependent variable: stress intensity factor *K*_1*c*_.

**Table 4:** The results of *p*-value estimation using the Dunnett’s test.

Composition	(*I*) *l/b*	(*J*) *l/b*	*p*-Value
CF0	0.3	0.2	1.000
	0.4	0.2	0.998
	0.5	0.2	0.466
CF25	0.3	0.2	0.847
	0.4	0.2	0.842
	0.5	0.2	0.585
CF50	0.3	0.2	0.749
	0.4	0.2	0.990
	0.5	0.2	0.589

**Table 5:** Average values of stress intensity factor *K*_1*c*_ [MPa·m^1/2^] for specimens with permanent *l/b* = 0.3 prepared by #3–#5 carbonization routes.

Composition	*l/b* = 0.3		
	Carbonization 3	Carbonization 4	Carbonization 5
CF0	0.71 ± 0.12	0.78 ± 0.09	3.80 ± 0.52
CF25	1.31 ± 0.58	1.28 ± 0.61	3.80 ± 0.27
CF50	1.51 ± 0.51	1.42 ± 0.36	3.95 ± 0.59

## Data Availability

The data used in this article can be retrieved by private corresponding author request.
